# Probing the Role of Digital Payment Solutions in Gambling Behavior: Preliminary Results From an Exploratory Focus Group Session With Problem Gamblers

**DOI:** 10.2196/54951

**Published:** 2024-07-23

**Authors:** Nathan Lakew, Jakob Jonsson, Philip Lindner

**Affiliations:** 1 Department of Clinical Neuroscience Karolinska Institutet Stockholm Sweden; 2 Center for Dependency Disorders Stockholm Health Care Services Stockholm Sweden

**Keywords:** digital payment solutions, online gambling behavior, sociotechnical, subjective experience, focus group

## Abstract

**Background:**

Technology has significantly reshaped the landscape and accessibility of gambling, creating uncharted territory for researchers and policy makers involved in the responsible gambling (RG) agenda. Digital payment solutions (DPS) are the latest addition of technology-based services in gambling and are now prominently used for deposit and win withdrawal. The seamless collaboration between online gambling operators and DPS, however, has raised concerns regarding the potential role of DPS platforms in facilitating harmful behavior.

**Objective:**

Using a focus group session with problem gamblers, this study describes a preliminary investigation of the role of DPS in the online gambling context and its influence on players’ gambling habits, financial behavior, choices of gambling environment, and the overall outcome of gambling subjective experiences.

**Methods:**

A total of 6 problem gamblers participated in a one-and-half-hour focus group session to discuss how DPSs are integrated into their everyday gambling habits, what motivates them to use DPS, and what shifts they observe in their gambling behavior. Thematic analysis was used to analyze the empirical evidence with a mix of inductive and deductive research approaches as a knowledge claim strategy.

**Results:**

Our initial findings revealed that the influence of DPSs in online gambling is multifaced where, on the one hand, their ability to integrate with players’ existing habits seamlessly underscores the facilitating role they play in potentially maximizing harm. On the other hand, we find preliminary evidence that DPSs can have a direct influence on gambling outcomes in both subtle and pervasive ways—nudging, institutionalizing, constraining, or triggering players’ gambling activities. This study also highlights the increasingly interdisciplinary nature of online gambling, and it proposes a preliminary conceptual framework to illustrate the sociotechnical interplay between DPS and gambling habits that ultimately capture the outcome of gambling’s subjective experience.

**Conclusions:**

Disguised as a passive payment enabler, the role of DPS has so far received scant attention; however, this exploratory qualitative study demonstrates that given the technological advantage and access to customer financial data, DPS can become a potent platform to enable and at times trigger harmful gambling. In addition, DPS’s bird’s-eye view of cross-operator gambling behavior can open up an opportunity for researchers and policy makers to explore harm reduction measures that can be implemented at the digital payment level for gambling customers. Finally, more interdisciplinary studies are needed to formulate the sociotechnical nature of online gambling and holistic harm minimization strategy.

## Introduction

As gambling became a readily available digital commodity, examining and regulating the influence of technology on gambling behavior has emerged as an important agenda for harm reduction research [1]. In the early days of the internet, Shaffer [2] noted that the innate nature of the “new” technology has the potential to become a potent vehicle to introduce certain biases in gambling, resulting in a shift in the subjective experiences of gamblers. Almost 30 years later, and with online gambling becoming commonplace, the extant literature has seemingly reached a consensus that technology-enabled gambling platforms with engaging and persuasive features can facilitate attention bias, impulsive betting, or longer time-on-devices, which can ultimately trigger or maintain addictive behavior [3].

The online gambling landscape has also created a demand for more enabling technologies, such as digital payment solutions (DPSs), for gambling activities. Under the umbrella of a general term known as financial technology, DPSs come in different forms, including digital wallets, payment gateways, or processors that can easily be integrated with online gambling sites or points of sale. Historically, the transfer and funding process of gambling has been perceived as cumbersome practicalities with unacceptable downtime in the eyes of gambling operators [4]. The fast adoption of DPS among the general population in other consumer domains (eg, digital shopping), its convenient account-to-account (direct bank) solution, and merchant-centric flexibility have quickly attracted gambling operators to integrate it into their digital platforms.

DPSs are seen as having a multifaceted advantage for the gambling industry [5]. First, DPS’s fast and seamless deposit and withdrawal solution facilitates longer gambling periods (ie, time-on-device) while solving the problem of downtime [4]. Second, DPS connects multimodal payment options to a one-click account [6]. Depending on local jurisdictions, a user can, for example, connect different payment modes, such as credit or invoice or several bank accounts, to their DPS account, to achieve multiple funding paths. Third, DPSs enable a unison provision of multiple services in one speedy process. For example, gambling operators can use DPS providers to both process payments and conduct Know-Your-Customer responsibilities of authenticating users [7], hence reducing the time and inconveniences associated with starting gambling. With the removal of lengthy registration and authentication processes, options, such as pay-and-play and no-account casinos, are emerging as the new standard of online gambling [8]. Other factors, including general familiarity with DPS in other e-commerce settings in past purchases and trust, can play a role in easy implementation, as well as the adoption of DPS in online gambling [9].

Apart from the technical aspect, DPS providers have benefited from recent policy relaxation of financial regulations, such as open banking [10]. Consequently, digital payment companies have the advantage of both agility and unprecedented access to financial data to influence consumer behavior (provided users’ consent). The nature of open banking allows more transparency and access to individual payment behavior; however, there is currently no strong incentive for DPS providers with gambling customers to be involved in the effort to promote responsible gambling (RG). Traditional policies and regulatory bodies that govern financial institutions, including DPS providers, mainly focus on ensuring that financial legal requirements are met and that a “well-functioning” marketplace is viable [11]. Financial decisions and well-being are broadly seen as individual responsibilities [12]. In addition, gambling policy making has historically focused on either RG measures implemented at gambling operator sites or consumers’ responsible behavior agendas.

Similar to other technological products, digital payments have been shown to influence subjective gambling experiences, even promoting harmful gambling. For instance, DPS enables an account-based registry in which consumers can connect different sources of funds (eg, multiple bank accounts) into a one-click account, which creates (an illusion of) “uninterrupted availability” of funds. Their fast-deposit mechanism and auto-deposit functionalities can result in lowering the awareness of money spent, leading to prolonged gambling consumption, and dark gambling flow—a state of intense immersion experienced by gamblers leading to a loss of awareness of money and time spent in gambling [13,14]. Previous studies have also theoretically explored DPS in the context of gambling, where payment technologies can be used to augment harm reduction measures [13].

Although the significance of DPS in gambling is beginning to gain attention [5,15-17], we are unaware of any past research that has examined players’ perspective of digital payment in an online gambling setting. In addition, and perhaps due to its interdisciplinary nature, there exists no conceptualization of the mechanism through which technology-enabled payment platforms can shift players’ subjective experiences toward harmful gambling behavior. As the current harm-minimization efforts in online gambling are heavily dependent on understanding, predicting, and promoting healthy gambling behavior, the research field needs to have a holistic understanding of how these tools are integrated into players’ everyday gambling experience and possibly facilitate harmful behavior.

This study aims to supplement the research area by conducting explanatory qualitative research involving first-hand accounts of DPS use among problem gamblers. Exploratory qualitative methods provide a practical approach for laying a strong foundation to further investigate emerging research areas [18]. In addition, qualitative methods, in general, are effective in understanding complex social phenomena of interest and human stories that involve probing participants regarding their experiences with open-ended questions [19]. In this study, the following explorative research questions were examined:

- How are DPSs used in gambling activities among problem gamblers, and what do they think about the influence of DPS on their gambling behavior?
- How can we conceptualize the role of DPS on the outcome of gambling behavior?

## Methods

### Ethical Considerations

The Swedish Ethics Authority approved this qualitative focus group study (DNR 2022-05843-01). All focus group participants gave informed consent before joining the sessions, and pseudonyms were used to protect their identities during the analysis. Participants were not compensated for their involvement in the study.

### Design and Data Collection

The research uses an exploratory qualitative design approach [20]. The approach provides methodological and theoretical flexibility to investigate topical research domains, aiming to identify important concepts and potential conceptual constructs [21,22]. Qualitative exploratory inquiries typically entail gathering primary data using techniques, such as interviews and focus groups, followed by a grounded theory approach analysis, occasionally leveraging on deductive insights from existing literature [23]. In this study, a focus group session serves as the primary data collection method. Following an explanatory research approach, the questions posed during the session were semistructured yet exploratory, providing participants with an opportunity to share their experiences freely.

Current studies on the adverse impacts of gambling indicate that harmful consequences, including financial ones, are prevalent in the problem gambling (PG) community [24]. Our study participants were selected from this group, allowing us to observe a wide range of harmful gambling behaviors. In addition, the homogeneity of the group has provided an environment where all participants felt comfortable to freely express their past experiences and views. However, it is important to note that individuals who do not have a gambling problem are not necessarily immune to the impact of DPS.

A total of 6 participants with a history of PG were recruited for a focus group interview. The participants were recruited through the Swedish National Association for Gambling Addicts—a national support group for problem gamblers. With the help of the support group administration, a recruitment leaflet was prepared and distributed during support group sessions. The leaflet outlined the goals of the focus group discussion and criteria for participating in the session: (1) experience in online gambling using 1 or more DPS, (2) having played in land-based gambling avenues using nondigital payment methods, and (3) being able to speak English. The participants accepted and returned the consent statement to the support group administration before the focus group session.

Participants were asked to complete brief questionnaires regarding demographics and gambling history before the focus group session started (Multimedia Appendix 1). The most common gambling modality reported was online gambling, including casinos and sports betting, with excitement chasing ranked as the main reason for gambling. The participants’ average gambling experience was more than 10 years. All participants were engaged in a biweekly support session. Table 1 provides supplementary profile details; however, following the ethical approval obtained and the sensitive nature of the research, we are only able to provide aggregate information regarding the participants.

**Table 1:** Participant characteristics.

Variables and options		Participants, n (%)
**Age (years)**		
	26-34	1 (17)
	35-50	3 (50)
	51-70	2 (33)
**Sex**		
	Male	5 (83)
	Female	1 (17)
**Marital status**		
	Married	2 (33)
	In relationship	2 (33)
	Separated	1 (17)
	Single	1 (17)
**Gambling type preference**		
	Land-based gambling	0 (0)
	Online gambling	4 (67)
	Both	2 (33)
**Experience of problem gambling (years)**		
	3-5	2 (33)
	7-10	1 (17)
	10+	3 (50)

### Procedure

The focus group discussion was conducted at the premises of the association after one of the support group sessions was completed. The focus group study format enables researchers to collect empirical evidence from multiple sources simultaneously while providing a sense of cohesiveness and a safe environment for participants to share their opinions [25]. In addition, live interaction between participants who share the same background (here, problem gamblers) provided an opportunity for honest and personal story discussions about their struggles and reflection on each other’s experiences. The first author (NL) led the focus group discussion, while the second author (JJ) played a supporting role during the meeting, including time management and follow-up questions. Additionally, the second author (JJ) participated as a professional psychologist with experience in gambling therapy to ensure safety in case the group discussions triggered any distressing memories among the participants.

The one-and-a-half-hour session started with an introduction of the aim of the focus group and the procedure. Participants were informed that they had the right to leave the session at any given time, refrain from responding to questions, and provide other relevant practical information. A semistructured set of questions was prepared in advance to conduct the discussion, and this material was used to organize the discussion around DPS’s influence on financial behavior, user gambling behavior, time spent on a device, gambling environment, and segment choices. The focus group protocol can be found in Multimedia Appendix 2.

### Analysis of Empirical Evidence

The focus group session was recorded and transcribed verbatim by NL. Thematic content analysis was then applied to analyze the material [26]. The analysis was conducted to develop a “ground-up” understanding of problem gamblers’ experience with DPS, which was later used to conceptualize the influence of DPS on the subjective experience of gambling. As such, thematic coding was performed using a grounded theory framework starting with “open coding” or manifest data analysis [27]. The transcribed focus group session was coded using QualCoder 3.2 (MIT-based open-source qualitative data analysis tool), with each participant assigned a pseudonym of P1-P6. The first 2 authors read and reread the empirical material for conversational-level open coding [28]. NL used the first coding, which was later reviewed by JJ for interpretation and coding validity. The manifest analysis followed an inductive approach where efforts were made to code block the material based on “first order value data,” which is what is obviously observable in the transcription [29]. With iterative coding and merging of codes at hand, a further validity review of codes was performed by JJ.

After organizing the merged codes into meaningful subthemes, potential themes emerged to categorize the subthemes. At the stage of building subthemes and themes, a deductive research approach was comparatively used as a lens to supplement theme building using existing relevant theoretical concepts in gambling and sociotechnical research, such as socio-materiality, imbrication, and reciprocal interaction theoretical frameworks [30-32]. The final themes and subthemes were then discussed by all 3 authors (NL, JJ, and PL) with the original text of transcription used as a confirmation of concept summary and naming before the final reporting.

## Results

### Overview

Three main themes emerged from the explorative analysis: (1) existing addictive needs dictate DPS’s “placement” in gambling, (2) DPS changes gambling subjective experience, and (3) problem gamblers’ perspectives on DPS. The first theme focuses on players’ way of integrating DPS into their gambling activities, covering three subthemes, each developing players’ appropriation of DPS into their gambling context to (1) workaround RG measures, (2) fit into gambling habit, and (3) shorter time to start and get a reward. With four subthemes, the second theme sheds light on DPS’s influence on gambling habits in the form of (1) subtle nudge on behavior, (2) opens up new gambling habits, (3) introduce bias, and (4) facilitate intangible qualities, such as trust to sanction different forms of illegal gambling.

Additionally, we find three subthemes with a nature of reflective content summarized in the third theme where problem gamblers react to DPS: (1) DPS is not for us, (2) pessimistic about RG tools in DPS, and (3) recommendations and opinions where the DPS role is conceived as having a minimal role in harmful gambling. Table 2 presents the analysis summary, and a more detailed theme development process can be found in Multimedia Appendix 3.

**Table 2:** Summary of themes and subthemes.

Main themes and subthemes			Summary of subthemes
**Existing addictive needs dictate DPS’s^a^ “placement” in gambling**			
	Workaround RG^b^ measures	DPS can be used to bypass RG tools. It can also enable players to maximize gambling “perks.”	
	Fit into gambling habit	DPS fits into a habit of a gentle start of the day with gambling. Gambling habits direct both the choices and ways of DPS use.	
	Shorter time to start and get a reward	DPS is used to make the “chores” of depositing “invisible.” DPS’s speedy withdrawal function is seen as a reward not just in terms of earning a prize but also for continuing to play.	
**DPS changes gambling subjective experience**			
	Subtle nudge toward harmful behavior	DPS can potentially facilitate dark flow in gambling. Subtle nudging toward longer “time on device” and harmful games, such as casinos.	
	Opens up new subjective experience	DPS enables one to develop new habits and ways of being a gambler. Rewards do not feel like wins anymore as they just represent numbers.	
	Introduces bias	DPS can introduce multiple biases, such as the illusion of control or the availability of unlimited funds. DPS can create a desensitizing feeling toward the value of money and gambling losses.	
	Facilitate intangible attributes	DPS can facilitate confirmation bias and increase trust in unregulated gambling sites.	
**Problem gamblers’ perspectives on DPS**			
	DPS is not for “us”	The integration of DPS into the gambling context needs scrutiny. The payment tool is used in gambling with an advantage for the industry in mind.	
	Pessimistic about RG tool in DPS	The effect of DPS on gambling behavior is minimal. Integrating “passive” RG tools, such as red warning with DPS, will not help problem gamblers.	
	Recommendations	Wins withdraw-focused RG measures in DPS can be more effective. DPS should be banned from operating in unregulated gambling sites.	

^a^DPS: digital payment solution.

^b^RG: responsible gambling.

### Existing Addictive Needs Dictate DPS Placement in Gambling

#### Workaround RG Measures

The participants noted that DPS provides opportunities to engage in gambling behaviors that would not be easily possible otherwise. In some instances, for example, participants reported that access to gambling was intact even though their names were included in the Swedish national self-exclusion registry because DPS enabled them to access unlicensed gambling operators. One participant described the use of DPS as a “negotiating system,” effectively offsetting the goal of RG tools implemented nationwide:

The bigger factor for players to go play on unlicensed (sites) is payment solutions. They (players) are in Spelpaus (self-exclusion registry). Therefore, they negotiate the ban with (a) payment solution in Sweden.

Participant 1

In other instances, some participants describe using DPS to circumvent specific RG features aimed at preventing harmful gambling, such as bonus offers and free spins on online casinos. Consequently, most participants recognized how DPS can constitute a ready-to-hand tool to overcome the “challenges” they face in satisfying gambling cravings and maximizing rewards.

Swedish casino is super easy, but I get (a) bonus in the unlicensed sites. Sometime(s) also find it cheaper to deposit in the unlicensed ones. I used the payment solution to do that. They (unlicensed sites) also offer free spins.

Participant 4

#### Fit Into Existing Gambling Habits

We also found players to appropriate DSP in line with their usual gambling habits. As they became comfortable with different payment solutions, they picked “favorites” that seamlessly fit into their gambling style. For example, 1 participant noted that his choice of a specific DPS—a popular Swedish mobile payment system—allowed him to start the day the way he “wanted it”:

I always start with the Swish [a mobile payment solution] on casinos, and when I reach my limits, I move on to the ones that I have my card saved and so on. For me, the easiest one to start the day with and then just push it forward.

Participant 4

Furthermore, the participant also mentioned that his choice of DPS has enabled him to make a smooth transition to other casino games after a win without worrying about lengthy fund transfer processes.

I want to change casino(s) because I’ve been lucky for a while and then I need to try the other sixteen so just moving around the money. Swish is the best one because if you played with the Swish, you could have the money quite quick(ly) back and move it forward.

Participant 4

The same sentiment was shared by other participants: different payment solutions were seen to satisfy different purposes and hence apt for different gambling routines.

#### Shorter Time to Start and Get a Reward

The most prominent reason for integrating DPS noted among all participants was related to the speed and “invisibility” a DPS affords in transferring funds during both deposit and win withdrawal. Most participants noted that they always wanted to quickly start gambling and perceived the process of depositing funds as a cumbersome chore to deal with before the fun started. DPSs that are already integrated with operators and commonly used among the players for other purposes were considered the best to make depositing “task invisible.” One participant noted that he chose a payment solution that makes betting and deposit almost synonymous:

When I’m thinking about payment solutions, for me I never used a solution where you had to make an account on one and then deposit. Everything should be direct. I never used the systems that were not directly connected to the account. Because it was too much of a hassle and then I had to see the money two times I would say (laugh). Yeah, but yeah, I had to see the money going in one then going away to another account. I didn’t want that.

Participant 5

Participants saw a speedy win withdrawal not just as receiving a prize but mainly as a resource to continue gambling. As such, win withdrawals may stay in the DPS account for fast redepositing. In addition, the need for speedy withdrawal seemed to correlate with the progressive worsening of PG.

The withdrawal time is important, especially when you need the money. When I started gambling, it was OK for me to wait for a couple of days. But when I got more and more addicted and intense, it became very important for me then. I remember I used to select Poker sites on stuff like how they looked; but that all went away once I got addicted, and payment solution and withdrawal time became important factors for me.

Participant 1

### DPS Changes Gambling Subjective Experiences

#### Subtle Nudges Toward Harmful Behavior

Most participants noted that DPS seamlessly fit into, and contributed to, the development of an uninterrupted “fictional world of gambling,” potentially affecting one’s financial well-being. Recounting such an experience, one participant described a dark flow of gambling where even getting a payday loan amid intense gambling activity was perceived as winning a jackpot:

You keep swishing; it is a fantasy world. When you are out, you (use) SMS loan money, it takes only 5 minutes. It’s as if you got a jackpot in (a) casino. It is the same feeling. It is a success as if you gamble and win.

Participant 5

In the same vein, DPS has been shown to subtly facilitate longer time-on-device by enabling operators’ actions, such as a suggestion to refill deposit accounts via a DPS one-click link and a sign-off. At times, refill amounts were suggested.

When money is almost gone, not completely gone almost gone always pops up “OK do you want to refill” ... that’s bad because then it’s like a quick link ... When you’re in a game and then it’s just OK, it feels quick and it’s like one press. That’s really stupid.

Participant 3

As noted in the first theme in passing, the speedy solution among payment methods was revealed to subtly nudge players toward harmful types of games. In many instances, participants were heard associating DPS with casino games where at times, the payment method is used as a reason for choosing gaming platforms.

You always choose those casinos that are easy to deposit. I used quick casinos to put small amounts of money many times ... I use the easiest, Swish, to deposit.

Participant 4

#### Open Up New Subjective Experience

In addition to subtle behavioral nudging, DPS can enable a new subjective experience of gambling behavior. Some participants reported that they developed new habits, such as gambling at workplaces or playing while driving as the adoption of DPS became widespread.

I started to play a lot more at (the) workplace when Swish came into (the) picture, and it was so easy. I have what you call blue-collar work that I must work (with) my hands, but I’ve seen when it was so easy you managed to work and play.

Participant 6

After 6 hours I could drive 300 km, and I use the easiest one; Swish to put the deposit while driving. One time I was about (to) crash: I panicked.

Participant 4

#### Introduces Bias

We also found some evidence of DPS’s nature in introducing multiple biases in gambling activities due to its technological makeup. In line with past research, the most obvious observation was the effect of digitalization on diminishing the value of money [33]. Some participants have also discussed how DPS enables them to connect different sources of funds (eg, different bank accounts, cards, or at times, invoices) into 1 DPS account to select deposit sources as they see fit. In addition to increasing money spent on gambling, such a setting created the illusion of unlimited funds during gambling activities.

They’re very easy because when I played, I had a few cards here which are used and they just picked up one of them, and (that) is OK.

Participant 4

You lose all the picture and it’s just a figure. You have money then out of money and done.

Participant 5

DPS was also shown to preface the illusion of control by enabling both a “controlled” amount of spending and frictionless payment. One participant noted that since his choice of DPS asks him to sign off each deposit amount, it felt like it was controlled spending. However, since the flow of signing off was frictionless and almost invisible, in practice, it was not so controlled.

For me, I used quick casinos to put in small amounts of money many times to not feel bad and lie to myself. I put 500 SEK again and again, but how many times? (laugh).

Participant 4

#### Facilitate Intangible Attributes

Finally, participants were heard discussing how DPS can influence their choice of games, gambling segments, and environment (ie, licensed vs unlicensed operators) by creating confidence and trust in the site where they are being integrated. The trust relegating effect was stronger for DPS, which is also used daily for other digital purchases.

It mattered to me if I knew the payment solution to play in the unlicensed sites, and it is a factor in selecting unlicensed sites.

Participant 2

### Problem Gamblers’ Perspectives on DPS

#### DPS Is Not for Us

The majority agreed that DPSs have negatively influenced their gambling behavior. There was a general sentiment that DPS are being swiftly integrated into gambling platforms without sufficient regard for the challenges they present to problem gamblers.

I think it’s scary that the Swish doesn’t have some red button. No one asks you if you do it again and again.

Participant 4

Yeah, or at least red flags ... The system wasn’t designed for us. It was designed for the general population.

Participant 1

Another reaction from the participants emphasizes that the DPS industry is taking advantage of the transition to a cashless society, which in general, gives an advantage to the financial industry.

#### Pessimistic About RG Tools in DPS

Some participants expressed pessimism about whether DPS-specific RG tools would even have helped them. More specifically, RG measures that are more reactive to players’ behavior, such as warnings or even bans based on gambling behavior, would not have helped. They noted that as long as the resource exists to spend on gambling, such measures can easily be evaded.

For me, the payment solution didn’t (matter), I mean, I would have gambled. It doesn’t matter if it took five minutes or ten, I would still have done it ... For the people who do it on a smaller scale then maybe it’s a little bit different. For me, it didn’t matter. If I just can get money in and out; that is the way, it is.

Participant 5

I’m just thinking that a limit or ban can be good in some ways in Fintech, but I also think that for me I wouldn’t stop there if there was a red button or something like that it wouldn’t have helped me.

Participant 6

#### Recommendations

Toward the end of the session, participants made a few proposals on how to make DPS a more RG-oriented product. This included measures concerning withdrawals, including making it harder to withdraw, removing cancel withdrawal options, and rethinking the design of win redepositing options. Other proposals include flagging deposit intensity as opposed to amount and the banning of DPS at unlicensed gambling operators.

It is easier to deposit than to withdraw your money, but it should be the other way around ... I will make it harder for withdrawal.

Participant 3

So, the amount might not be a good sign, but how many times I did (deposit) should be the sign for them to signal ... no one asks you if you do it again and again.

Participant 4

## Discussion

### General Findings

Our preliminary findings revealed DPS’s ability to seamlessly integrate into existing habits and enhance players’ ability to achieve their goals. We also found evidence that the harmful influence of DPS can, at times, originate from its technological makeup. Consequently, changes in subjective experiences that emerged in the use of DPS were multifaceted. First, existing gambling routines, sensemaking of DPS features, and variation of individual habits and behaviors influence how and to what extent DPS affects gambling behavior. This aligns with existing literature that shows how various users interpret and use DPS features in diverse ways. Some problem gamblers, for example, were shown to perceive DPS’s budget tools as a means to address their gambling issues while players who identify themselves as healthy gamblers considered such features as merely a “safety net” [5] or found them restrictive and unnecessary [17].

Second, our findings indicate that the nature of DPS can influence players’ gambling behaviors in a variety of ways. For instance, participants felt that they were in control of their spending since they authorized the deposit “button” at all times, while the frictionless payment flow diminished the money spent. As such, the inherent flexibility and “invisibility” of DPS in players’ gambling experiences enabled the mediation of conflicting values and facilitated impulsive deposits over rational decision-making. In another respect, we observed a more pervasive nature of DPS where it can trigger new gambling behaviors. For instance, participants reported gambling more often in situations that they would not typically gamble in (eg, workplace or driving), while others noted that they developed trust in illegal operator sites because of their familiarity with the DPS used by those operators.

Finally, participants’ discussion on their general perception of DPS surfaced different perspectives that span from an unfavorable view of DPS to an agnostic attitude to recommendations on how to make payment solutions better. The findings underline players’ keen awareness of digital payment pitfalls in their gambling behavior and disappointment in the lack of RG effort in the area. In what follows, some theoretical and practical implications of this study are outlined.

### Theoretical Implications

This study highlights the need for interdisciplinary research to conceptualize the role of DPS in gambling activities. On the one hand, the evidence of how existing gambling behavior influences players’ way of integrating DPS for gambling is in line with research that underscores the “social imperative on the technical”—the argument that technological features or their use are dependent on, and a product of human choices [34]. Typically, characterized as a sociocentric framing, users and their existing habits influence how technology is used, hence the outcome of subjective experience [35]. From this perspective, DPS can be seen as a tool to enhance one’s ability to work around, augment, or enable existing habits in gambling activities.

On the other hand, the findings that emphasize DPS’s both subtle and pervasive influence in determining the outcome of gambling, underscore a techno-centric perspective where technology has a more “deterministic” demeanor and direct effect on subjective experience. From this contrasting perspective, a more dominating role can be manifested where DPS can constrain, institutionalize, or directly shape players’ gambling activities. In addition to being visibly deterministic, technology can have an “imposter” nature by presenting itself as a response to human needs while subtly nudging players toward harmful experiences [36]. With so-called user-centric design, for instance, gambling technologies have been shown to trigger greater betting intention, an illusion of control, and attention bias [3,37]. In both the “subtle and pervasive” nature of technology influence, our analysis has shown that DPS can facilitate gambling dark flow, longer time-on-device, access to unsafe gambling environments, diminish the value of spending, and nudge toward harmful games, such as casinos. Finally, the innate nature of technology, such as its transparency, is associated with lessening the pain of paying, resulting in impulsive consumption [33].

**Figure 1:**
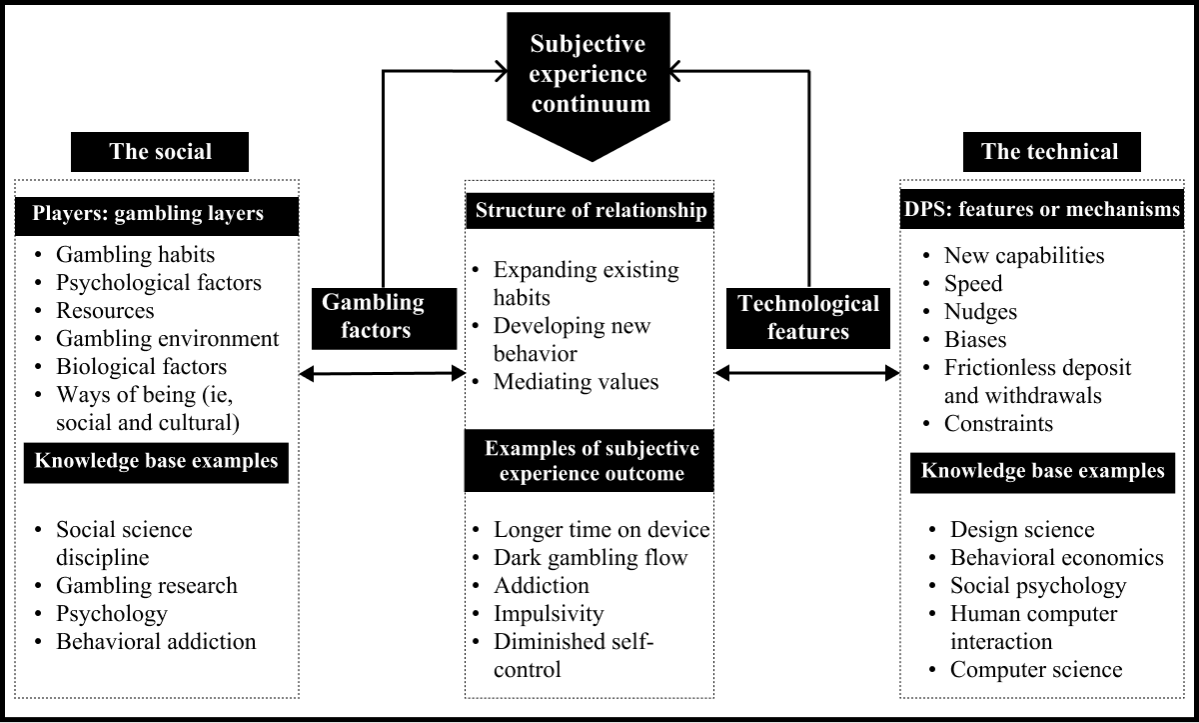
Preliminary conceptual framework to examine the influence of DPS in gambling subjective experience. DPS: digital payment solution.

As such, conceptualizing the influence of DPS on gambling behavior constitutes a confluence of multifactorial structures. Put another way, capturing the interplay between the socio and technical aspects, symbolized by the hyphen that connects the ‘socio-technical’ term, is imperative to conceptualize the role of DPS in gambling activities [38]. The societal context on the left side of the hyphen encompasses cultural factors, sociodemographics, psychology, and broader societal influences, all of which shape habits and behaviors [39]. Representing the technical, the right side of the hyphen encompasses DPS’s technological features and mechanisms that facilitate players in achieving their goals or directly enable new capabilities and forms of gambling activities. A preliminary conceptual framework illustrating the interaction between the social and technical aspects within the context of DPS is depicted in Figure 1. DPS-related changes in gambling subjective experiences (eg, addiction) can be seen as an outcome of various factors from the social and the technical. In terms of their scale of influence, these factors are not equally distributed and vary based on contexts, such as individuals and time (eg, severity of gambling addiction).

As players integrate DPS into their gambling activities in the context of existing habits, their subjective experience can shift toward harmful gambling based on what the technology affords them to achieve. In addition, the framework suggested that layers of contextual factors that can affect an individual’s use of DPS in gambling are best developed in social disciplines, such as gambling research, psychology, and behavioral addiction [39]. DPS can also directly influence gambling experience outcomes in the form of technological features using mechanisms, such as nudging, pervasive enframing, and constraining options. These features can introduce harm to gambling activities in the form of speed and biases or enable new ways of harmful gambling, which in turn can be translated to longer time-on-device, excessive spending, or dark flow of gambling. The development of these features and mechanisms can be both technical and psychological; hence, they apply design and behavioral science principles.

### Practical Implications

Historically, intervention measures, policies, and RG tools predominantly focus on gambling operators since they are responsible for how the gambling product is ultimately presented to the customer. However, the findings from this study illustrate that DPS can play an active role in facilitating harmful gambling experiences. Account-level protection, such as a “universal” budget limit and single customer view options with high spending and gambling intensity data sharing, are some of the opportunities that exist for policy-anchored implementations. In addition, it is important to detect and disable technology-enabled workarounds, such as gambling on unlicensed sites to rein in DPS’s offsetting nature of RG efforts. In line with policies, effective design interventions can also be used to compensate for “harmful” affordances. For instance, as noted by the participants, intentional delays in win withdrawal and monthly gambling spending reports across operators are within reach for any financial institution given their data set.

Finally, there have been calls for a more comprehensive and cross-sectoral approach, incorporating measures, such as data interoperability among industry stakeholders and integrating public health perspectives [40,41]. With appropriate regulations, DPS with gambling customers can play a pivotal role in data-sharing efforts that enable an overview of a customer’s gambling activities across different operators. In addition, public health preventive measures, such as affordability testing, which takes into account both financial state and gambling behavior, can be implemented in DPS platforms as a deterrent against unaffordable gambling [42]. These preventive measures also serve as a “safety net” for nonproblem gamblers, thereby facilitating the implementation of a population-oriented harm minimization strategy [5].

### Limitations and Future Research

Given the exploratory nature of this study, several limitations have been identified, highlighting both the necessity for further investigation and new research opportunities. First, due to its limited size of empirical evidence, there is a need for more studies with a larger number of participants for the generalizability of this study’s results. Second, we decided to select problem gamblers as focus group participants, as financial decision-related gambling harm is more visible among high-intensity gambling user groups. However, the associations made between gambling behaviors and DPS can benefit from future diverse groups of gambling participants. Furthermore, there were no notable demographic-based differences observed in the use of DPSs, possibly due to the group’s uniformity in terms of payment methods used, types of gambling, or the regular attendance of participants in biweekly gambling support sessions. We also want to stress that the proposed conceptual framework presented here should be seen as a preliminary effort to start the conversation of how DPS, in its capacity as a technological product, can potentially facilitate harmful behavior. Using the corresponding knowledge base noted on either side of the hyphen, future research can identify and conceptualize key sociotechnical elements and their mode of interaction in shaping players’ behavior.

Finally, technological products can be intentionally designed to mediate certain values—positive interventions or self-interest goals—that can shift subjective experiences for better or worse [43]. As such, more research is needed on the design aspect of technology that has a target audience in gambling. Due to space limitations and the scope of this paper, we will not go further into the design research field. However, the concepts of user-centered design, design for appropriation, persuasive system design methods, and overall intentional design research need to be part of future research to examine technological antecedents and their influence on gambling behavior [44-48].

### Conclusions

Given that online gambling will continue to optimize its edge with the support of technological products, new challenges are emerging for researchers and policy makers in the effort to reduce gambling harm. This study focused on problem gamblers’ first-hand experience of DPS to examine its role in the overall setup of online gambling activities. Our preliminary findings suggest that seamless collaboration between operators and DPSs can create a potential environment conducive to facilitating harmful gambling behavior. Given their central place in gambling activities, however, DPS providers are also in a unique position to implement umbrella-like harm reduction measures, such as limit settings applicable across gambling sites. As such, there exist avenues for policy makers to onboard DPS to positively contribute to the effort of the RG agenda, as they have a bird’s-eye view of gamblers across different operators.

Finally, as digital payment providers continue to attune their products to gambling merchants using “user-centric” design and consumer behavior data, the field needs to examine DPS’s platform-like role in transforming players’ subjective experiences in online gambling. This study highlights possible interdisciplinary avenues, such as sociotechnical-oriented research, to fully grasp the role of technological products in general and DPS in particular in gambling behavior. The preliminary framework demonstrates the interplay between the sociotechnical components—from privileging the social while acknowledging the technical to giving equal agency for both sides of the hyphen to tech-dominated social experiences. More research is needed to further delineate the structure of the relationship between enabling technological products and the characteristics of gamblers that will ultimately influence the outcome of gambling.

## Appendix

Multimedia Appendix 1 Brief demographics and gambling background questionnaires.

Multimedia Appendix 2 Focus group material: summary of discussion questions.

Multimedia Appendix 3 Empirical material analysis and theme development process.

## Abbreviations

DPS: digital payment solution
PG: problem gambling
RG: responsible gambling

## Data Availability

The data sets generated and analyzed during this study are not publicly available due to the sensitivity of the data and per ethical approval guidelines.

## References

[1] Cemiloglu D, Naiseh M, Catania M, Oinas-Kukkonen H, Ali R (2021). The fine line between persuasion and digital addiction.

[2] Shaffer HJ (1996). Understanding the means and objects of addiction: technology, the internet, and gambling. J Gambling Stud.

[3] Flayelle M, Brevers D, King DL, Maurage P, Perales JC, Billieux J (2023). A taxonomy of technology design features that promote potentially addictive online behaviours. Nat Rev Psychol.

[4] Schüll ND (2012). Addiction by Design.

[5] Lakew N (2022). "Show me the money": preliminary lessons from an implementation of intervention tools at the payment gateway level. J Gambl Stud.

[6] Rao S.R. (2015). E-wallet-a 'pay'volution.

[7] Kiayias A, Kohlweiss M, Sarencheh A (2022). Peredi: privacy-enhanced, regulated and distributed central bank digital currencies.

[8] Gustafsson T (2021). Designing a European gambling self-exclusion registry. Tallinn University of Technology.

[9] Archer N, Wang S, Pei Y (2021). Reputation, familiarity and use intention for e-payment services: a comparison of pure-play and click-and-mortar e-payment services. IJSTM.

[10] Farrow GS (2020). Open banking: the rise of the cloud platform. J Paym Strategy Syst.

[11] Baker TH, Stone C (2020). Making outcomes matter: an immodest proposal for a new consumer financial regulatory paradigm. SSRN J.

[12] (2017). Financial well-being: What it means and how to help. Consumer Financial Protection Bureau.

[13] Gainsbury SM, Blaszczynski A (2020). Digital gambling payment methods: harm minimization policy considerations. Gaming Law Rev.

[14] Lavoie RV, Main KJ (2019). When losing money and time feels good: the paradoxical role of flow in gambling. JGI.

[15] Ghaharian K, Abarbanel B, Kraus SW, Singh A, Bernhard B (2023). Players gonna pay: characterizing gamblers and gambling-related harm with payments transaction data. Comput Human Behav.

[16] Limbrick-Oldfield EH, Chua C, Cringle N, MacDonald K, Ferrari MA, Zhang K, Clark L (2021). Cashless gambling and the pain of paying: effects of monetary format on slot machine gambling. Addict Res Theory.

[17] Swanton TB, Tsang S, Collard SB, Garbarino E, Gainsbury SM (2024). Cashless gambling: qualitative analysis of consumer perspectives regarding the harm minimization potential of digital payment systems for electronic gaming machines. Psychol Addict Behav.

[18] Swedberg R (2020). Exploratory research. The Production of Knowledge Enhancing Progress in Social Science.

[19] Farrelly P (2013). Choosing the right method for a qualitative study. Br J School Nurs.

[20] Stevens RE, Loudon DL, Cole H, Wrenn B (2013). Exploratory (qualitative) research. Concise Encyclopedia of Church and Religious Organization Marketing. 1st Edition.

[21] Stebbins RA (2001). Exploratory Research in the Social Sciences.

[22] Rendle KA, Abramson CM, Garrett SB, Halley MC, Dohan D (2019). Beyond exploratory: a tailored framework for designing and assessing qualitative health research. BMJ Open.

[23] Mansourian Y (2008). Exploratory nature of, and uncertainty tolerance in, qualitative research. New Libr World.

[24] Langham E, Thorne H, Browne M, Donaldson P, Rose J, Rockloff M (2016). Understanding gambling related harm: a proposed definition, conceptual framework, and taxonomy of harms. BMC Public Health.

[25] Onwuegbuzie AJ, Dickinson WB, Leech NL, Zoran AG (2009). A qualitative framework for collecting and analyzing data in focus group research. IJQM.

[26] Morgan DL (1993). Qualitative content analysis: a guide to paths not taken. Qual Health Res.

[27] Corbin JM, Strauss A (1990). Grounded theory research: procedures, canons, and evaluative criteria. Qual Sociol.

[28] Myers G (2006). ‘Where are you from?’: identifying place. J Socioling.

[29] Cash P, Snider C (2014). Investigating design: a comparison of manifest and latent approaches. Des Stud.

[30] Cecez-Kecmanovic D, Galliers RD, Henfridsson O, Newell S, Vidgen R (2014). The sociomaterialty of information systems: current status, future directions. MIS Q.

[31] Goh JM, Gao G, Agarwal R (2011). Evolving work routines: adaptive routinization of information technology in healthcare. ISR.

[32] Leonardi PM (2011). When flexible routines meet flexible technologies: affordance, constraint, and the imbrication of human and material agencies. MIS Q.

[33] Mazar N, Plassmann H, Robitaille N, Lindner A (2016). Pain of paying? A metaphor gone literal: evidence from neural and behavioral science. Rotman School of Management.

[34] Sarker S, Chatterjee S, Xiao X, Elbanna A (2019). The sociotechnical axis of cohesion for the IS discipline: its historical legacy and its continued relevance. MIS Q.

[35] de Guinea AO, Markus ML (2009). Why break the habit of a lifetime? Rethinking the roles of intention, habit, and emotion in continuing information technology use. MIS Q.

[36] Feenberg A (2008). Critical theory of technology: an overview. Information Technology in Librarianship: New Critical Approaches.

[37] Newall PWS (2022). Reduce the speed and ease of online gambling in order to prevent harm. Addiction.

[38] Meyer ET, Fichman P, Rosenbaum F (2014). Examining the hyphen: the value of social informatics for research and teaching. Social Informatics: Past, Present and Future.

[39] Hilbrecht M, Baxter D, Abbott M, Binde P, Clark L, Hodgins DC, Manitowabi D, Quilty L, SpÅngberg J, Volberg R, Walker D, Williams RJ (2020). The conceptual framework of harmful gambling: a revised framework for understanding gambling harm. J Behav Addict.

[40] Wardle H, Reith G, Langham E, Rogers RD (2019). Gambling and public health: we need policy action to prevent harm. BMJ.

[41] Ladouceur R, Blaszczynski A, Shaffer HJ, Fong D (2016). Extending the Reno model: responsible gambling evaluation guidelines for gambling operators, public policymakers, and regulators. Gaming Law Rev Econ.

[42] Marionneau V, Ruohio H, Karlsson N (2023). Gambling harm prevention and harm reduction in online environments: a call for action. Harm Reduct J.

[43] Cemiloglu D, Arden-Close E, Hodge SE, Ali R (2023). Explainable persuasion for interactive design: the case of online gambling. J Syst Software.

[44] Abras C, Maloney-Krichmar D, Preece J (2004). Encyclopedia of Human-Computer Interaction.

[45] Dix A (2007). Designing for appropriation.

[46] Goodman E, Stolterman E, Wakkary R (2011). Understanding interaction design practices.

[47] Gregory J (2003). Scandinavian approaches to participatory design.

[48] Torning K, Oinas-Kukkonen H (2009). Persuasive system design: state of the art and future directions.

