# Supplementary material for: Probing the Role of Digital Payment Solutions in Gambling Behavior: Preliminary Results From an Exploratory Focus Group Session With Problem Gamblers
Source: JMIR Hum Factors. 2024 Jul 23;11:e54951. doi: 10.2196/54951 (PMC11303895; doi:10.2196/54951)
Supplement: Multimedia Appendix 1 [file humanfactors_v11i1e54951_app1.docx]

1. Age

18 – 25  26 – 35  35 – 50  51 – 70  71+

2. Gender

Man  Woman  Others  Don’t want to say

3. Marital status

Married  In relationships  Separated/divorce  Widowed  Single

4. Employment status

Full-time employed  Part-time  Unemployed  Retired  Disability welfare

5. Length of time having a gambling problem (before or now)

Less than 12 months.  1–2 years  3 – 5  7 – 10  10+

6. How long have you been gambling?
 Less than 12 months.  1–2 years  3 – 5  7 – 10  10+

7. Generally, what is your main goal/s of gambling?

Just for fun by myself  Extra income  Socialization with friends  Trilled feeling

8. Which of the following gambling games do you prefer or play?

Slots and table games  Poker  Sport betting  Horse racing  Bingo  Lottery
 Fantasy sports

9. Which of the following gambling type do you prefer most?

Land based gambling  Online gambling  Both
