# Supplementary material for: Probing the Role of Digital Payment Solutions in Gambling Behavior: Preliminary Results From an Exploratory Focus Group Session With Problem Gamblers
Source: JMIR Hum Factors. 2024 Jul 23;11:e54951. doi: 10.2196/54951 (PMC11303895; doi:10.2196/54951)
Supplement: Multimedia Appendix 2 [file humanfactors_v11i1e54951_app2.docx]

- In general, what do you think about the pros and cons of using digital payment solutions in the context of your gambling habit?
- Was it possible for you to use different payment methods and/or connect different sources of funds? If so, did it affect your gambling or financial behavior in any form?
- In particular, how does digital payment method do you think affects your deposit and withdrawal behavior?
- Do you recall any situation that might have affected your use of RG tools as a result of digital payment solutions?
- Did the method of payment affect your choices of gambling operators and/or the selection of games?
- Did the payment solution enable/oblige you to do things in online gambling that wouldn’t be possible otherwise? Discuss.
- Why are the reasons for selecting a particular digital payment solution provider over the others?
- Do you see any effect of digital payment in your gambling activities in terms of financial behavior, personality, and attitude to money spent in gambling?
- If you were invited to make changes to digital payment solutions in gambling, what would that be?
- Of all the things we discussed, what to you is the most important?
- Is there anything else that you think we missed to discuss today regarding digital payment solutions in gambling context?
