# Supplementary material for: Probing the Role of Digital Payment Solutions in Gambling Behavior: Preliminary Results From an Exploratory Focus Group Session With Problem Gamblers
Source: JMIR Hum Factors. 2024 Jul 23;11:e54951. doi: 10.2196/54951 (PMC11303895; doi:10.2196/54951)
Supplement: Multimedia Appendix 3 [file humanfactors_v11i1e54951_app3.docx]

**Table S1.** *Theme: Existing addictive needs dictate DPS’s ‘placement’ in gambling* **–** subthemes, sample codes, and quotes

| **Workaround RG measures** | **Fit into gambling habit** | **Shorter time to start and get a reward** |
| --- | --- | --- |
| **Gambling after being blocked**  *The bigger factor for players to go play on unlicensed (sites) is payment solutions. They (players) are in Spelpaus (blocked list). So, they negotiate the ban with payment solution in Sweden. (P1)* | **Gambling on different providers**  *I want to change casino because I’ve been lucky for a while and then I need to try the other sixteen so just moving around the money. Swish is the best one because if you played with the Swish, you could have the money quite quick back and move it forward you never put them in your bank account to just play. (*P4) | **Faster deposit time**  *When I'm thinking about payment solutions, for me I never used a solution where you had to make an account on one and then deposit. Everything should be direct. I never used the systems that weren’t directly connected to the account. Because it was too much of a hassle and then I had to see the money two times I would say (laugh). Yeah, but yeah, I had to see the money going in one then going away to another account. I didn't want that. (P5)* |
| **Bypass limit setting tools**  *During the pandemic, they set limits on site. If I meet the limit, I'll just switch to other sites. It is easier with a payment solution. I always change the amount that I could lose ‘OK, now it's X amount, OK yeah but now we're down to X amount OK; I'll go down to the other amount and then the other amount. It didn't matter if there was a ban or if I meet (hit) my limits or so, it didn’t matter for me. (P5)* | **Easing into gambling**  *I always start with the Swish on casinos, and when I reach my limits, I move on to the ones that I have my card saved and so on. For me, the easiest one to start the day with and then just push it forward*. (P4) | **Shorter win withdrawal**  *The withdrawal time is important, especially when you need the money. When I started gambling, it was OK for me to wait for a couple of days. But when I got more and more addicted and intense, it became very important for me then. I remember I used to select Poker sites on staff like how they looked; but that all went away once I got addicted, and payment solution and withdrawal time became important factors for me. (P1)*  *I used PayPal because they had a shorter withdrawal time than a regular card. so if I use my regular card like my visa it took me maybe a week or 10 days to get the money back. the potential win. But with PayPal, it took 24 hours. I used that for many years*. (P1) |
| **Chasing bonus/reward**  *Swedish casino is super easy, but I get bonus on the unlicensed sites. Sometime, also find it cheaper fee to deposit in the unlicensed ones. I used the payment solution to do that. They (unlicensed sites) offer free spins.* (P4)  **. . .**  *So, the free spin is like a trigger for people to gamble more*. (P3) |  |  |

**Table S2.** *Theme: DPS changes gambling subjective experience* **–** subthemes, sample codes, and quotes

| **Subtle nudge toward harmful behavior** | **Opens up new subjective experience** | **Introduces bias** | **Facilitate intangible attributes** |
| --- | --- | --- | --- |
| **Facilitate dark flow**  *You keep swishing, it is a fantasy world. When you are out, you (use) SMS loan money, it takes only 5 minutes. It's as if you got a jackpot in a casino. It is the same feeling. It is a success as if you gamble and win.* (P5)  *I'm an emotional player if I lose, I get tilted really tilted they just put more and more money all the time but at the land casino, you have to go to the ATM or something, and could happen that I cool it down a bit. I am a gambling addict. Online, I just yes to 50, 100, 1000, or 10,000 … I can't turn it down; the money basically is gone.* (P6) | **‘New’ way of being**  *I started to play a lot more at workplace when Swish came into picture, and it was so easy. I have what you call blue-collar work that I must work (with) my hands, but I've seen when it was so easy you managed to work and play.* (P6) | **Illusion of unlimited fund**  *They’re very easy because when I played, I had a few cards here which is used and they just picked up one of them, and (that) is OK.* (P4)  *Yeah, I had three connected to payment solution, but probably more, I think. I don't think they have limitations*. (P5)  *I know sometimes you could play and get an invoice, is it still like that? That was the worst*. (P2) | **Enable trust on unlicensed sites**  *Yes, it mattered to me if I knew the payment solution to play in the unlicensed sites, and it is a factor in selecting unlicensed sites*. (P2) |
| **Time on device**  *When money is almost gone not completely gone almost gone always pops up ‘OK do you want to refill’ didn't want to deposit, that's bad because then it's like a quick link. When you started OK, I've to click 1 2 3 times you go whatever. But I mean, when you're in a game and then it's just OK, it feels quick and it's like one press. That's really stupid.* (P3) | **Gambling on the go**  *I put 500 SEK again and again; but how many times (laugh). After 6 hours I could drive 300km, and I use the easiest one; Swish to put the deposit while driving”. One time I was about to crash; I panicked.* (P4) | **Illusion of control**  *For me, I used quick casinos to put in small amounts of money many times to not feel bad and lie to myself. I put 500 SEK again and again, but how many times? (laugh).* P4 | **Select a gambling site based on Fintech**  *I think it's more critical to choose a gambling site based on payment solutions. As the more gambling addict you are, (the more) this becomes a bigger hurdle; the speed is the issue here.* (P1) |
| **Harmful reinforcement**  *And sometimes it even suggests the sum that you should put on.* (P2)  . . .  *I'm getting charged, yeah, but the sum couldn't be less you had to put in the minimum amount and if you want to deposit less than that then you have to log out, so it was easier to use a bigger amount and because it's that was super easy. It's just one click.* (P4)  **Casino is the new normal**  *You always chose those casinos that are easy to deposit . . . I used quick casinos to put small amounts of money many times . . . I use the easiest, Swish, to deposit*. (P4) | **Wins don’t feel like wins**  *When you went to a casino you used to get happier for those kinds of wins compared to online where you have the numbers basically when you go to just a number.* (P1) | **Diminish the value of money and win feelings**  *It's so quick to make the swish so you just gambled, and you don't check your account until it's there's no money left just to keep on swishing.* (P4)  *We have been I would say desensitized from what cash is for many, many years . . . So, money is like lost its value and then losing the feeling of having this thick bunch on your hand.* (P2) |  |

**Table S3.** *Theme: Problem gamblers’ perspectives on DPS* – subthemes, sample codes and quotes

| **DPS is not for ‘us’** | **Pessimistic about RG tools in DPS** | **Recommendations** |
| --- | --- | --- |
| **No protection for problem gamblers**  *I think it's scary that the Swish doesn't have some red button. No one asks you if you do it again and again.* (P4)  . . .  *Yeah, or at least red flags*. (P1) | **It doesn’t matter … when you gamble big**  *For me, the payment solution didn’t (matter), I mean, I would have gambled. It doesn't matter if it took five minutes or ten, I would still have done it . . . For the people who do it on a smaller scale then maybe it's a little bit different. For me, it didn't matter. If I just can get money in and out; that is the way, it is*. (P5) | **No. of deposit matters**  *So, the amount might not be a good sign, but how many times I did(deposit) should be the sign for them to signal … no one asks you if you do it again and again.* (P4) |
| **DPS adoption in gambling needs scrutiny**  *The system wasn't designed for us. It was designed for the general population, and for those, there are not as many setbacks but for us who are gambling addicts of course it's going to be a problem*. (P1) | **I don’t think a warning could help**  *I'm just thinking that a limit or ban can be good in some ways in Fintech, but I also think that from my standpoint I wouldn't stop there if there was a red button or something like that it wouldn't have helped me*. (P6) | **Win withdrawal target RG tools**  *It is easier to deposit than to withdraw your money, but it should be the other way around . . . I will make it harder for withdrawal.* (P3)  *I would say, I don’t know if it is possible, but when you withdraw you have to fill a form such as if you are thinking of depositing again.* (P4)  . . .  *Also, ban the cancel withdrawal option. (P3)* |
| **DPS is more advantageous for banks than players**  *I agree, you have a big advantage using DPS in general. But also, the thing that that never you never talked about is to whom mostly. When we started with the cashless society or Fintech everything was OK because cash in hand it's unsafe it's bad, but the real reason is actually because the handling of the money costs money for the banks a lot. so, it's not like it's not an advantage for us but it's also it's a disadvantage and you don't see both sides. I would say that has trickled over to the gambling*. (P5) |  | **Ban DPS on unlicensed sites**  *Make it hard to play in no licensed casinos using these payment methods.* (P2) |
